# The association of maternal-infant interactive behavior, dyadic frontal alpha asymmetry, and maternal anxiety in a smartphone-adapted still face paradigm

**DOI:** 10.1016/j.dcn.2024.101352

**Published:** 2024-02-03

**Authors:** Edyta Swider-Cios, Elise Turk, Jonathan Levy, Marjorie Beeghly, Jean Vroomen, Marion I. van den Heuvel

**Affiliations:** a Department of Cognitive Neuropsychology, Tilburg University, Warandelaan 2, 5000 LE, Tilburg, the Netherlands; b Department of Neonatology, University Medical Center Utrecht, Utrecht University Heidelberglaan 100, 3584 CX, Utrecht, the Netherlands; c Department of Criminology and Gonda Brain Research Center, Bar-Ilan University, Ramat-Gan, 5290002 Israel; d Department of Neuroscience and Biomedical Engineering, Aalto University, Rakentajanaukio 2, 02150, Espoo, Finland; e Department of Psychology, Wayne State University, 5057 Woodward Ave, Detroit, USA

**Keywords:** Synchrony, EEG hyperscanning, Frontal alpha asymmetry (FAA), Maternal anxiety

## Abstract

Mother-infant interactions form a strong basis for emotion regulation development in infants. These interactions can be affected by various factors, including maternal postnatal anxiety. Electroencephalography (EEG) hyperscanning allows for simultaneous assessment of mother-infant brain-to-behavior association during stressful events, such as the still-face paradigm (SFP). This study aimed at investigating dyadic interactive behavior and brain-to-behavior association across SFP and identifying neural correlates of mother-infant interactions in the context of maternal postnatal anxiety. We measured frontal alpha asymmetry (FAA), a physiological correlate of emotion regulation and a potential marker of risk for psychopathology. To emulate real-life interactions, EEG and behavioral data were collected from 38 mother-infant dyads during a smartphone-adapted dual-SFP. Although the behavioral data showed a clear still-face effect for the smartphone-adapted SFP, this was not reflected in the infant or maternal FAA. Brain-to-behavior data showed higher infant negative affect being associated with more infant leftward FAA during the still-face episodes. Finally, mothers with higher postnatal anxiety showed more right FAA during the first still-face episode, suggesting negative affectivity and a need to withdraw from the situation. Our results form a baseline for further research assessing the effects of maternal postnatal anxiety on infants’ FAA and dyadic interactive behavior.

## Highlights

• Dual-EEG was used to enable simultaneous measurement of neural activity in mother-infant dyads during natural interaction.
• Infants showed a behavioral still-face effect in smartphone-adapted episodes, but the neural data did not reflect this pattern.
• Infants’ data indicated associations between behavioral states and frontal alpha asymmetry (FAA) in the still-face episodes.
• Mothers with higher postnatal anxiety showed more right FAA during the first still-face episode.
• The results contribute to understanding the neural and behavioral processes underlying mother-infant social exchange.

## 1 Introduction

Early mother-infant interactions are foundational for the development of children’s socioemotional and cognitive skills (Wade et al., 2022; Atzil et al., 2011). The ideal outcome of early dyadic face-to-face interactions is the achievement of mutual regulation and positive social exchange (Feldman, 2012, Miller et al., 2002, Swingler et al., 2014). During social exchanges, mother and infant temporally match their interactive behavior, emotion, and biology, described as synchrony (Feldman, 2007). Appropriate caregiver responses to infants’ signals, such as crying or smiling, promote mother-infant contingent responsivity and facilitate the development of infants’ self-regulatory skills, which are needed to cope with emotionally difficult situations (Leerkes et al., 2009, Perry et al., 2016, Swingler et al., 2014). One way to study early dyadic interactions is the Still Face Paradigm (SFP; (Tronick et al., 1978), which creates a stressful situation for mothers and infants. During the SFP, mother-infant dyads are observed during a sequence of face-to-face interaction tasks consisting of a normal interaction episode, during which mothers and infants interact as they normally would, followed by a still-face episode during which mothers become unresponsive and maintain a neutral facial expression, and ending with a resumption of normal interaction (reunion) (Tronick et al., 1978). In a meta-analytic review of the SFP literature, infants exhibit a robust still-face effect during the still-face episode of the SFP, compared to their behavior during the baseline interaction episode, characterized by a decrease in positive affect (e.g., less smiling) and an increase in negative affect (e.g., increase in fussing) (Mesman et al., 2009). A carry-over of negative affect and partial rebound of positive affect are also observed during the reunion episode, in comparison to infants’ behavior during the baseline episode. Other investigators have utilized a double SFP to heighten infants’ physiological reactions, which includes 5 successive episodes: baseline, still-face 1, reunion 1, still-face 2, and reunion 2 (DiCorcia et al., 2016).

Individual differences in infants’ responses to the maternal still-face are also reported and are associated with maternal sensitive responsiveness to the infant during the baseline episode of the SFP or in other contexts (Gunning et al., 2013, Moore et al., 2009, Tarabulsy et al., 2003). Infants whose mothers were more sensitive with them in previous interactions produced more frequent attempts to re-engage the still-faced mother and more positive affect, whereas infants who had experienced less maternal sensitivity exhibited greater distress and disengagement (Braungart-Rieker et al., 2014). Although numerous studies using the SFP have assessed links between maternal sensitive responsiveness during mother-infant interaction and infants’ responses to the maternal still-face, relatively few studies have investigated the association between dyadic interactive behaviors and dyadic brain responses (Behrendt et al., 2020, Perone et al., 2020).

Recently, EEG has been applied in a simultaneous recording of brain activity with two or more participants, which is referred to as dual-EEG or EEG hyperscanning (Babiloni et al., 2007, Goldstein et al., 2018, Perone et al., 2020; for review see: Turk et al., 2022). This innovative paradigm shift in EEG enables the simultaneous measurement of the neural activity of both mother and infant during mother-infant interaction (Turk et al., 2022). EEG hyperscanning has the potential to provide insight into inter-brain dynamics and synchronization during dyadic interactions, which can help with better comprehension of (co-)regulatory processes and brain-to-behavior associations (Atzaba-Poria et al., 2017). Inter-brain synchrony could be driven by a shared sensory input, as well as internal cognitive processes supporting social interaction and communication, while the degree of social connectedness coordinates enhanced inter-brain synchronization (Djalovski et al., 2021, Turk et al., 2022). In a recent study, Perone et al. (2020) applied EEG-hyperscanning to investigate how SFP influences mother-infant frontal alpha asymmetry (FAA) and how differences in mother-infant interaction quality are reflected in dyadic brain activity. FAA is a neural correlate of emotion regulatory processes, measured as the difference between left and right hemisphere frontal activation in the alpha frequency band (Killeen and Teti, 2012, Smith et al., 2017, Swingler et al., 2014). More left frontal alpha activity is associated with positive emotional states and approach motivation, whereas more right frontal activity is associated with negative emotional states and avoidance (Coan et al., 2006, Davidson, 1993, Davidson, 2004, Perone et al., 2020). Changes in FAA can be reflected across SFP as it becomes more stressful for mothers and infants over time, with more right FAA in dyads as the SFP progresses due to increased regulatory demands (Perone et al., 2020).

The aims of our study were threefold. The first aim was to explore dyadic behavior and FAA across SFP episodes. Here, we utilized a double SFP including two still-face episodes and two reunions to increase the regulatory demands across the whole procedure (DiCorcia et al., 2016, Haley and Stansbury, 2003). We did not take away toys from the child during the still-face episode and we applied a smartphone-adapted version of maternal behavior during the still-face episode of the SFP to make the task more ecologically valid (Konrad et al., 2021, Stockdale et al., 2020). Asking mothers to look at a smartphone with a neutral face during the still-face episode is consistent with many parents’ behavior in daily life, as parents commonly use screen-based media during childcare (Barr et al., 2020, Kildare and Middlemiss, 2017; Myruski et al., 2018; Swider-Cios et al., 2023). We expected to observe a still-face effect for infants in both the behavioral and neural data and for mothers in neural data. For the behavioral data, we expected to see increased levels of infant negative and flat affect, lower levels of positive affect, and more object engagement during the still-face episode as compared to infants’ behavior during the baseline and reunion episodes (H1a). Regarding the neural data, we hypothesized that both infants and mothers would exhibit more right frontal activity during the still-face episodes than during the normal interaction episodes (H1b). Additionally, we hypothesized that dyads would share a pattern of frontal alpha activity (synchrony). Or, in other words, mothers’ and infants’ FAA would be correlated over the duration of the task as the task induces increasingly more stress and desire to withdraw for both mothers and infants (H1c).

The second aim was to examine dyadic brain-to-behavior associations during the SFP. Here, we hypothesized that infant positive affect (H2a) and negative affect (H2b) during the SFP episodes would be related to maternal and infant FAA. Specifically, we hypothesized that infants with higher negative and positive affect would show more rightward and leftward FAA respectively. For mothers, we hypothesized that mothers would have more leftward FAA in case of higher infant negative affect. Positive and negative affect have been linked to FAA markers, with the former being associated with relative left frontal EEG activation and the latter with right FAA (Atzaba-Poria et al., 2017, Palmiero and Piccardi, 2017). Since maternal sensitivity was shown to be associated with infant FAA (Hane and Fox, 2006, Swingler et al., 2014) we further hypothesized that higher maternal sensitivity, defined as mother’s ability to perceive, interpret, and appropriately respond infant’s signals (Ainsworth et al., 1974), during the baseline and reunion episodes, would be related to more leftward infant FAA (H2c).

The third aim was to identify the neural bases of mother-child interaction during the SFP in the context of maternal postnatal anxiety. The quality of mother-infant interactions can be affected by several factors, including maternal depression and anxiety. Compared to research on maternal postnatal depression (for review see Slomian et al., 2019), literature describing the association between maternal postnatal anxiety on dyadic interactive behaviors and neural responses is sparse, and the reported results on interactive behaviors are mixed (Kaitz and Maytal, 2005, Smith et al., 2022). Anxious mothers tend to show less affection and more controlling behavior with their infants during social interactions (Nicol-Harper et al., 2007, Ostlund et al., 2017). This parenting style may mediate the association between maternal anxiety and infants’ withdrawal from challenging tasks (Ostlund et al., 2017). We therefore expected that a rightward shift in mother-infant FAA during the SFP would be more pronounced when mothers had higher anxiety scores (H3a). Additionally, we explored possible interactions between dyadic brain-to-behavior associations and maternal postnatal anxiety (H3b). Here, we hypothesized that the brains of mothers with higher postnatal anxiety would be more affected by infants’ negative affect during the still-face episodes. Given the importance of establishing neural correlates of mother-infant interactions and examining them in more naturalistic conditions, the findings of this study may form a baseline for further research on the impact of maternal anxiety on mother-infant interactive behavior.

## 2 Methods

This study was a part of the Brains in Sync Project and was carried out in a research lab in the Department of Cognitive Neuropsychology at Tilburg University, The Netherlands. The study was approved by the Ethical Review Board of Tilburg University, The Netherlands (number: RP34). No pre-registration was done for this study.

### 2.1 Participants

The mother-infant dyads that participated in this study were recruited via social media (e.g., Facebook), newsletters, and flyers on the campus and (baby) stores. Before the lab visit, mothers and their partners (if applicable) signed informed consent forms to participate in the study. The participants were compensated for their time in the form of a small present for the infant. Only dyads with infants up to 12 months of age were included in this study. Characteristics of mothers and infants included in this study are presented in Table 1.

**Table 1:** Participant characteristics.

Participant	N	M (SD)	Min	Max
**Mothers**	35			
Age at EEG measurement (years)		32 (6.43)	27	42
Education				
MBO (middle-level applied education)	2			
HBO (higher professional education)	12			
University	13			
Higher academic (Ph.D./PostDoc, etc)	8			
SCL-90_Anx score	35	13.51 (4.55)^a^	10	32
PSAS score	35	73.97 (12.41)^b^	53	102
**Infants**	36			
Age at EEG measurement (months)		9.16 (1.3)	5.72	11.74
Sex				
Girl	19			
Boy	17			
IBQ-vsf Negative Affectivity	36	24.42 (8.82)	6	55

Notes. SCL-90 = Symptom Checklist. PSAS = Postpartum Specific Anxiety Scale. ^a^ In a Dutch population sample, scores between 12-14 are considered “mean anxiety”, scores between 15-22 are considered “above average and high anxiety” and scores of ≥ 22 as “extremely high anxiety” (Arrindell and Ettema, 2003, Arrindell and Ettema, 2003). ^b^ Initial validation of the English-language version proposed a cut-off score of 112 or above for possible detection of clinical levels of anxiety (Fallon et al., 2016).

Overall, 41 infants participated in this study. Three of the 41 dyads were excluded from further analysis. For one dyad, the child was already 13 months. The second mother-infant pair was excluded because the dyad was not able to complete the SFP due to the infant’s distress. The third dyad was excluded due to a technical error, resulting in a lack of reliable data. An additional three mothers and two infants were excluded from further statistical analyses as more than 10% of their electrodes were interpolated. Finally, for FAA analysis 30 mothers (35 with partial data) and 29 infants (36 with partial data) had complete data from all SFP episodes. Overall, 35 mothers (with a mean age of 32 years; SD = 6.43) and 36 infants (with a mean age of 9.16 months years; SD = 1.3; 19 girls, 52%; 72% of infants were between 7–9 months) were included in the main analyses involving FAA data. For behavioral analysis and coding, 33 dyads had complete data from all SFP episodes (38 partial data). Missing data were not imputed.

### 2.2 Procedures

#### 2.2.1 Online questionnaire

Prior to the lab visit and EEG measurements, mothers were asked to fill out a 7-part online questionnaire using Qualtrics software (Qualtrics, Provo, UT, www.qualtrics.com). This questionnaire took approximately 60 min to complete and included questions about maternal, but not paternal, demographics (e.g., maternal age, nationality marital status, education level, employment status, and health), childcare arrangements, infant temperament (Infant Behavioral Questionnaire – Very Short Form (IBQ-vsf; (Putnam et al., 2014)maternal anxiety (SCL-90 Anxiety subscale and Postpartum Specific Anxiety Scale, PSAS), dispositional mindfulness (Mindful Parenting; (de Bruin et al., 2014)), and mothers’ history of childhood maltreatment (Childhood Trauma Questionnaire, CTQ; (Bernstein et al., 2003)). Only data from the demographics, infant temperament, and maternal anxiety questionnaires were evaluated in the current study.

#### 2.2.2 Maternal anxiety

Maternal anxiety was measured using the validated Dutch version of the anxiety subscale of the SCL-90 and the PSAS. The SCL-90 is a multidimensional questionnaire developed to screen for a range of psychological symptoms on nine subscales, including anxiety. The SCL-90 has good validity (Arrindell and Ettema, 2003, Arrindell and Ettema, 2003). The anxiety subscale included in the current study consists of 10 items addressing symptoms that are associated with manifest anxiety. Mothers rated different items, e.g., ‘Feeling of fear or panic’, on a 5-point scale where 1 = not at all and 5 = extremely. The PSAS evaluates the frequency of anxiety experienced by mothers during the first postpartum year (Silverio et al., 2021). This scale consists of 51 items that measure the frequency of maternal and infant-oriented anxieties experienced during the previous week. Mothers rated various statements, e.g. ‘I have had negative thoughts about the relationship I have with my baby’, using a 4-point Likert scale, where 1 = not at all and 4 = almost always. The PSAS has been described as a valid and reliable research tool for assessing anxiety (Fallon et al., 2016). Each measure was used separately in further analysis.

#### 2.2.3 Dual-EEG measurements

**Fig. 1:**
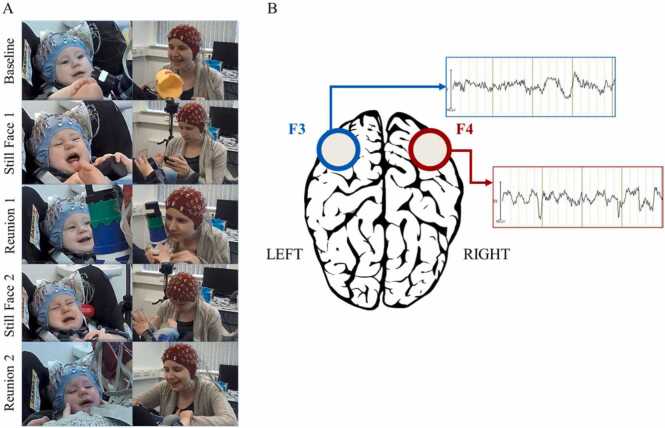
Mother-infant interactions and frontal alpha asymmetry (FAA). (A) Dual-EEG of mother-infant dyad during smartphone-adapted Still-Face Paradigm (SFP). (B) The assessment of differences in the activation between left and right hemispheres during frontal alpha measurements. Note: The mother gave consent to use images presented on panel A.

Recruited mother-infant dyads were invited to come to the Life Span Laboratory for a 1.5-hour visit during which the 30-minute EEG measurements were done. EEG measurements were performed on mothers and their infants using two connected BioSemi Active Two systems with 64 channels (for further information about this hyperscanning set up, please see (Barraza et al., 2019, Turk et al., 2022). The electrodes were placed using the 10–20 system and the sampling rate was set at 512 Hz. During the EEG measurements, mothers sat in a chair facing their baby, who was seated in an infant seat (Fig. 1A). The EEG was synchronized by adding an event marker in the EEG for both mothers and their infants using Eprime.

#### 2.2.4 Double still face paradigm

EEG recording was divided into 2-minutes segments, corresponding to the five phases of the double SFP: normal baseline interaction, still face 1, reunion 1, still face 2, and reunion 2 phase (Fig. 1A). During the baseline and reunion episodes, mothers were instructed to engage in free, natural play with their infants. During the still-face periods, mothers were instructed to look at the black screen of their smartphones and not make any form of interaction (neither verbal nor non-verbal) with their infants and be emotionally unavailable. The smartphone adaptation of the maternal still-face episode was utilized to increase the ecological validity of the study (Barr et al., 2020, Konrad et al., 2021, Myruski et al., 2018). Furthermore, toys were not removed during still-face episodes.

#### 2.2.5 EEG processing

The EEG data were then analyzed using Brain Vision Analyzer (Brain Products; www.brainproducts.com/) separately for mothers' and infants’ data. Alpha power was computed for the 6–9 Hz frequency band for infants, and 8–13 Hz for adults (Perone et al., 2020). The 6–9 Hz frequency band is the dominant frequency for infants this age (Saby and Marshall, 2012). The measurement of frontal alpha frequency was of interest, as this frequency is present throughout the lifespan, with specific values changing during development. EEG data for each participant were examined and bad channels were removed or interpolated (via Formula evaluator) to reconstruct missing EEG channels. If more than 10% of electrodes were interpolated, those participants were excluded from further analyses. A new reference was manually applied by taking an average of all channels, including interpolated channels. This was done separately for mothers' and infants’ data. Independent Component Analysis (ICA) was run on the mothers’ data to remove eye blink artifacts. ICA was not performed on infant EEG data, as ICA has limitations when processing infant EEG data due to the less stereotypical nature of infant movements as compared to adults (Fujioka et al., 2011, Georgieva et al., 2020, Noreika et al., 2020). Furthermore, the characteristics of eye movement artifacts in infant EEG data can change as infants develop, making detection and removal of such artifacts a challenge when infants with broader age range participate. There are studies describing new algorithms that can be potentially applied to infant EEG data for artifact removal (Marriott Haresign et al., 2021). However, those were trained on data of infants with smaller age gaps and may not be suitable for the data in the current study. Additionally, in a recent publication, Delorme (2023) argued that EEG data is ”better left alone” and that ICA removal of eye movements did not improve data quality.

Data from two frontal electrodes, F3 and F4 from mothers and infants were extracted for further analysis. The resulting data were high-pass filtered at 1 Hz, low-pass filtered at 30 Hz, and a 50 Hz notch filter was applied. Next, data were divided into segments representing each SFP phase, followed by the division into 2 s bins with 50% overlap. Data were then corrected for artifacts via artifact rejection with criteria for amplitude set at −200 µV (min) and 200 µV (max), for gradient set at 80 µV/ms, for interval length set at 200 ms, and maximum allowed absolute difference set at 150 µV. The time-frequency decomposition on the epochs was done using Fast Fourier Transform (FFT). The remaining data were included in the subsequent analyses if at least 20 segments per condition were present.

For mothers, on average, 111.6 segments (93%; SD = 10.0) were retained in the baseline condition, 116.2 segments (97%; SD = 16.3) in the still face 1 condition, 112.2 segments (94%; SD = 9.1) in the reunion 1 condition, 102.2 segments (85%; SD = 42.4) in the still face 2 condition, and 99.7 segments (83%; SD = 37.2) in the reunion 2 condition. For infants, on average, 111.7 segments (93%; SD = 14.5) were kept in the baseline condition, 104.7 segments (87%; SD = 19.6) in the still face 1 condition, 104.3 segments (87%; SD = 24.0) in the reunion 1 condition, 89.0 segments (74%; SD = 44.2) in the still face 2 condition, and 96.0 segments (80%; SD = 37.3) in the reunion 2 condition. The computation was done for each SFP phase by subtracting natural log-transformed alpha at left site F3 from right site F4 (Perone et al., 2020), from mothers and infants separately. As alpha power is inversely related to brain activity, higher alpha asymmetry scores indicate a relatively greater left frontal brain activity (Coan and Allen, 2004, Zhao et al., 2018; ). The scores of the recorded data are at zero, positive, or negative, which reflects frontal symmetry, relatively greater left frontal activity, or relatively greater right frontal activity, respectively (Briesemeister et al., 2013, Smith et al., 2017).

#### 2.2.6 Behavior coding

During the EEG measurements, three video cameras were used to record the behavior for further analysis (one camera focused on the mother’s face; one on the baby’s face; one on the overall interaction). To operationalize qualitative dimensions of maternal, infant, and dyadic behavior during the double SFP, ten rating scales from the MACY Infant-Parent Coding System (Earls et al., 2009)were used. One scale evaluated maternal behavioral sensitivity, five scales assessed maternal affect (positive affectivity, negative affectivity, anxiety, and warmth), one scale assessed infant object engagement, and three scales evaluated infant affect (positive affectivity, negative affectivity, and flat affect/withdrawal). Each dimension was scored using a 5-point Likert scale ranging from 1 = “none” to 5 = “very high”). Behavior and affect were coded based on the frequency of occurrence and for how long they were observed for. For example, an infant that smiled and showed interest in an interaction during the whole SFP episode would be coded as 5 for positive affect in that episode. Maternal sensitivity was scored only during the three dyadic interactions (i.e., baseline episode and both reunion episodes). Each scale tapped the level and quality of each behavioral dimension, and scale points included discrete behavioral and vocal cues and facial expressions. For instance, smiles, laughter, interest, and clapping were behaviors included in the positive affect scale, and sad and angry facial expressions, limb flailing, crying, whining, and fussing were included in the negative affect scale. A lack of these expressions (e.g., a blank gaze) and social withdrawal were included in the flat affect/withdrawal scale. Here, only the ratings of maternal affective sensitivity, infant object engagement, and infant affect were used.

Two independent, trained coders rated these dimensions of infant, maternal, and dyadic behavior from videotapes of the double SFP. Intercoder reliability was very good to excellent (ICCs ranged between 0.896 – 0.982, see Table S1 in Supplementary Information for detailed scores).

### 2.3 Covariates

#### 2.3.1 Demographic factors

Infant sex and maternal education were evaluated as potential covariates in the current study. Although results are inconsistent (Mesman et al., 2009), infant sex has been linked to variations in parent-infant interaction quality (de Mendonça et al., 2019, Feldman, 2003, Weinberg et al., 1999). Furthermore, maternal education has been described as a potential mediator in frontal asymmetry (Tomarken et al., 2004). Finally, as alpha power changes across infancy Marshall et al. (2002)), and given a broader age range of infants included in the study, infant age was also evaluated as a potential covariate Information on infants’ age, biological sex and maternal education was obtained from the demographic’s questionnaire.

#### 2.3.2 Infant temperament

Previous research showed that variations in infant temperament are linked to mother-infant synchrony (Quiñones-Camacho et al., 2020), and maternal brain responses (Killeen and Teti, 2012, Kuzava and Bernard, 2018). Here, infant temperament was assessed using the Dutch version of the Infant Behavioral Questionnaire – Very Short Form (IBQ-vsf) (Gartstein and Rothbart, 2003, Putnam et al., 2014). The IBQ-vsf includes 37 items assessing three temperament dimensions in infants between ages 3 and 12 months: Surgency, Negative Affectivity, and Effortful Control (Gartstein and Rothbart, 2003). Mothers rate different aspects of their infants’ behavior during the past 7 days using a 7-point scale (1 = never, 7 = always), e.g., ‘When your baby was tired, how often did he/she seem upset?”. Mothers were able to choose the “Does not apply” answer if their infants have not shown a certain type of behavior. In this study, only the Negative Affectivity subscale was included in the statistical analyses.

### 2.4 Statistical analyses

Statistical analyses were done using IBM SPSS Statistics 29 software. First, the mean and standard deviation for all the study variables were calculated. Then, differences in maternal and infant FAA scores by infant sex were analyzed. These results are reported in Table S2 and Table S3 of the Supplementary Information but are not further discussed as they are not the focus of the study.

To test the first (H1a – changes in infant behavior across the five episodes of the SFP) and second (H1b – changes in dyadic FAA across the five SFP episodes) hypotheses, two repeated-measure ANOVAs were performed (one per hypothesis). To see if dyads share a pattern of frontal alpha activity (H1c), a correlation was run between mother and infant FAA during different SFP episodes.

Next, to examine whether infant behavioral states during the still-face episodes were related to maternal FAA (H2a and H2b), Spearman correlation analyses were carried out between infant positive and negative affect scores and maternal and infant FAA scores across all episodes of the SFP. Furthermore, to test whether maternal sensitivity was related to infant FAA (H2c), a second Spearman correlation was run between maternal affective sensitivity scores and infant FAA during the baseline and both reunion episodes.

To test the third hypothesis of whether maternal anxiety impacts maternal and infant FAA (H3a), a Spearman correlation was performed between SCL-90 anxiety, PSAS, and dyadic FAA scores. Next, multiple, hierarchical regression analysis was performed for each SFP episode separately (5 regression analyses were run separately for mothers and infants) to check for covariates. As no correlation between SCL-90 and FAA scores was found, only PSAS scores were added to the model as an independent variable. Mother FAA was used as the dependent variable in the first model and infant FAA was used as the dependent variable in the second model. The following covariates were entered into each model: infant age, infant sex, maternal education, and infant Negative Affectivity (IBQ-vsf).

Finally, to explore possible interactions between dyadic brain-to-behavior associations and maternal postnatal anxiety (H3b), an interaction between maternal PSAS scores and infant negative affect was added to the previous regression analyses. Specifically, maternal PSAS scores and infant negative affect were entered as predictive variables, as well as an interaction between these two variables on maternal FAA.

### 2.5 Sensitivity analyses

Several sensitivity analyses were performed to ensure that our results were not biased by data quality or analytic decisions. For the first sensitivity analysis, we rerun the analyses with FAA scores excluding outliers found in maternal FAA data. Outliers were identified in SPSS using the “extreme outlier” definition of 3 *IQR (interquartile range). For the second sensitivity analysis, we reran our analyses including only mothers (*N* = 29) and infants (*N* = 29) with complete data from all SFP episodes. In the third analysis, we ran the analyses using the SCL-90 cut-off scores and PSAS median scores (two groups, with high and low scores). Finally, a fourth sensitivity analysis was done to test the influence of mutual movements on the results. We tested whether the number of available segments per condition was associated with FAA scores to assess whether FAA scores were potentially influenced by dyadic movement on the signal. Here, the number of available segments per condition was taken as a proxy for movement and data quality, where more artifacts in the data lead to less segments, while more segments would indicate higher quality of data and less overall movement.

## 3 Results

### 3.1 Descriptives

Participant characteristics are presented in Table 1. Education level was homogenous among the study mothers (92% had a professional-level education or higher). Sixty-eight percent of mothers were either married or in a registered partnership. Mothers’ average score on the SCL-90 anxiety subscale was 13.51 (SD = 4.55), with seven mothers having above-average scores suggesting high anxiety, and two mothers having scores above 22 suggesting extremely high anxiety. However, the scores on the PSAS scale indicated that none of the mothers experienced clinical levels of postpartum-specific anxiety (scores ranging from 53 to 102). We also observed a significant, strong correlation between maternal anxiety on the SCL-90 and the PSAS (*r* = .712, *p* < .001, *N* = 38). Finally, higher maternal PSAS scores were associated with higher infant Negative Affectivity (*r* = .326, *p* = .046, *N* = 38).

### 3.2 Changes in affective behavior during SFP

**Fig. 2:**
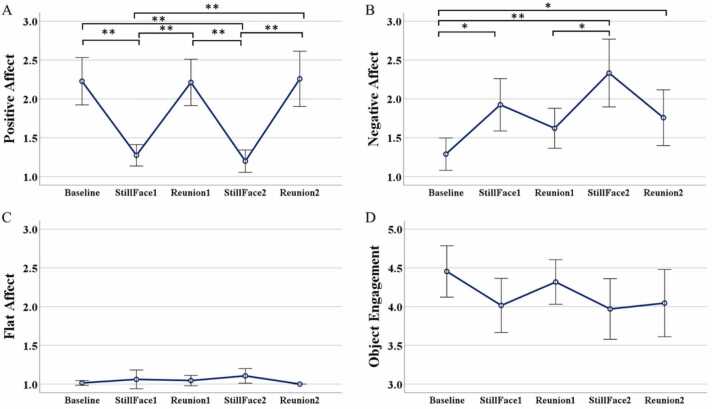
Changes in infant’s affect and object engagement across SFP. Significant changes in positive (A) and negative (B) affect across the SFP were observed. Results for flat/withdrawn affect (C) and object engagement (D) showed no significant changes across the baseline, still face, and reunion episodes. *p < .05 * * p < .001 Error bars ± 2 SE.

For our first hypothesis (H1a), we examined changes in infant affective behavior across the SFP to examine whether the classic still-face effect could be observed in this sample. Based on individual infant scores from behavioral coding, we observed that 68% of infants showed a still face effect when it comes to positive affect, while 53% of infants showed a still face effect for negative affect. The overall results showed that infant affective behavior changed in expected ways during the SFP (Fig. 2A & B). In particular, infant’s positive (*F* (3.07, 98.27) = 19.12, *p* < .001) and negative affective behavior (*F*(3.06, 97.97) = 8.9, *p* < .001) changed significantly across the SFP episodes consistent with a still-face effect. Additionally, there was a greater decrease in positive affective behavior during both still episodes than during the interaction episodes of the SFP (*p* < .001, Sidak corrected). Infant negative affect also increased during the first (*p* = .014, Sidak corrected) and second (*p* = .006, Sidak corrected) still-face episodes, relative to the interaction episodes. Interestingly, we observed significantly higher negative affect in the final reunion phase of the SFP as compared to the baseline episode (*p* = .048, Sidak corrected), suggesting that infants did not return to baseline levels of negative affect.

In contrast, analysis of infant flat/withdrawn affective behavior showed a non-significant increase during both still-face episodes in comparison to the interaction episodes (*p* > .05, Sidak corrected) (Fig. 2C). We also observed small, non-significant changes in infant object engagement across the episodes of the SFP (*p* > .05, Sidak corrected), with a small decrease in object engagement during both still-face episodes (Fig. 2D).

### 3.3 Dyadic frontal alpha asymmetry during SFP

Mean FAA scores for both mothers and infants across all SFP episodes are presented in Table 2. Overall, FAA scores showed that 21 out of 36 infants (58%) and 20 out of 35 mothers (57%) exhibited, on average, more relative right frontal activity during the whole SFP. Six infants showed a shift from left to right frontal activity during the first still-face episode, whereas, for the second still-face episode, five infants showed a similar shift. Here, only one infant showed such a shift in both still face episodes. For mothers, four mothers showed a shift from left to right frontal activity in both still face episodes. For two mothers the shift from left to right frontal activity was observed only in the first still face episode, while one mother showed such a shift only in the second still face episode. The repeated-measures ANOVA did not show any significant differences in mother or infant FAA between the interaction and still-face episodes (H1b). Additionally, we did not observe any correlation between maternal and infant FAA scores (H1c; Table S4 in Supplementary Information).

**Table 2:** Mother and infant FAA scores across each SFP phase.

Participant	N	M (SD)	Min	Max
Mother FAA				
Baseline	35	.057 (.48)	-.77	1.49
Still Face1	35	.052 (.40)	-.44	1.42
Reunion 1	35	.048 (.53)	-1.00	1.99
Still Face 2	30	.013 (.34)	-.57	1.03
Reunion 2	31	.015 (.36)	-.76	.86
Infant FAA				
Baseline	36	-.019 (.21)	-.41	.40
Still Face1	36	-.016 (.17)	-.41	.33
Reunion 1	35	-.011 (.18)	-.35	.30
Still Face 2	30	-.012 (.22)	-.28	.68
Reunion 2	32	-.012 (.22)	-.41	.40

### 3.4 Brain-to-behavior associations during SFP

The second aim of our study was to explore brain-to-behavior associations during the SFP. There were no significant correlations for the associations between infant positive affect and mother-infant FAA (H2a) (Table 3). A different pattern of results was observed for infant negative affect (H2b): Significant positive correlations were found between infant negative affect and infant FAA scores during the first still-face episode (*r* = .424, *p* = .010, N = 36) and second still-face episode (*r* = .491, *p* = .006, N = 30). These results indicate more leftward (positive) FAA scores and approach motivation when infants show higher infant negative affect (H2b). For maternal FAA scores, we detected positive correlations between infant negative affect and maternal FAA during the baseline episode (*r* = .357, *p* = .035, N = 35), also indicating more leftward (positive) FAA scores for higher infant negative affect. Additionally, we looked at possible correlations between infant FAA and maternal behavior during the baseline episode and both reunion episodes (H2c). Here, we observed a significant negative correlation between maternal affective sensitivity and infant FAA scores during the baseline episode (*r* = −.434, *p* = .008, N = 36), indicating more rightward (negative) FAA scores in infants of mothers that showed more affective sensitivity during dyadic interaction.

**Table 3:** Infant and maternal FAA correlations with infant positive and negative affect during SFP, and maternal sensitivity during baseline and reunion episodes.

Correlation	Baseline	Still Face 1	Reunion 1	Still Face 2	Reunion 2
Infant FAA x Infant positive affect	-.289	-.160	-.074	-.333	-.216
Infant FAA x Infant negative affect	.133	.424*	.301	.491**	-.138
Mother FAA x Infant positive affect	-.277	-.265	-.027	-.110	-.165
Mother FAA x Infant negative affect	.357*	.204	-.019	.000	-.202
Infant FAA x maternal sensitivity	-.434**	-	-.062	-	-.292

**Correlation is significant at the 0.01 level (2-tailed)

*Correlation is significant at the 0.05 level (2-tailed)

### 3.5 The role of maternal postnatal anxiety

**Fig. 3:**
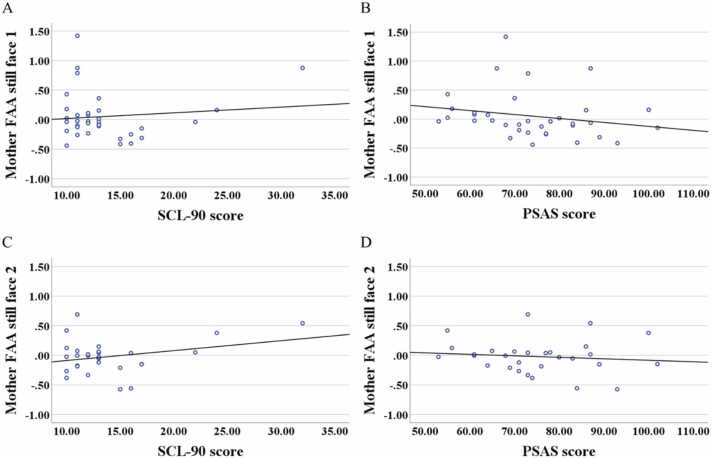
Spearman correlations maternal FAA scores and scores on anxiety scales. Correlation between maternal SCL-90 scores and maternal FAA scores in the first (A) and second (C) still face episodes. Correlation between maternal PSAS scores and maternal FAA scores in the first (B) and second (D) still face episodes. Note: Fitted lines (based on Pearson correlations) for the graphical purpose only.

Our third aim was to identify the neural bases of mother-infant interactions in the context of maternal postnatal anxiety. We expected that the rightward shift in mother-infant FAA during SFP would be more pronounced in the case of higher maternal anxiety scores (H3a). Results of correlational analysis (Fig. 3) revealed a significant negative correlation only between maternal PSAS score and maternal FAA during the first still-face episode (*r* = −.380, *p* = .024, *N* = 35), but not for the second still-face episode (*r* = −.187, *p* = .322, *N* = 30). No significant correlation was detected between maternal anxiety on the SCL-90 and maternal FAA in the first (*r* = −.189, *p* = .276, N = 35) or second (*r* = −.014, *p* = .942, N = 30) still-face episodes. The results also did not reveal any significant associations between infant FAA scores and maternal scores on the anxiety scales (Table S5**.** in Supplementary Information). Furthermore, maternal FAA across the SFP was not significantly predicted by maternal anxiety on the PSAS (Table S6 in Supplementary Information).

Finally, we expected to see interactions between dyadic brain-to-behavior associations and maternal postnatal anxiety (H3b). However, in the multiple regression analyses, we did not detect any significant interaction effects between maternal postnatal-specific anxiety and infant affective behavior on maternal FAA during the first still-face (*β* = .822, *p* = .46) or the second still-face episode (*β* = −.230, *p* = .87) (Table S7 in Supplementary Information).

### 3.6 Sensitivity analysis

For the first sensitivity analysis, we rerun the analyses where we excluded the two detected outliers in maternal FAA samples. Removal of the outliers reduced the magnitude of the negative correlation between postnatal-specific anxiety scores and maternal FAA in the first still-face episodes to statistical non-significance (*r* = −.333, *p* = .058). Additionally, the positive correlation between infant negative affect and maternal FAA during the baseline episode also became statistically non-significant (*r* = .236, *p* = .186).

For the second sensitivity analysis, we reran our analyses including only mothers and infants with complete data for all SFP episodes. Complete data (all SFP phases) were available for 29 mothers and 29 infants. In the analysis of brain-to-behavior associations (Table S8 and Table S9 in Supplementary Information), we detected an additional significant positive correlation between infant negative affect and infant FAA scores during the first reunion episode (*r* = .396, *p* = .034), which suggests more leftward infant FAA in case of higher infant negative affect scores. Furthermore, an additional significant negative correlation was observed between infant positive affect and maternal FAA during the baseline episode (*r* = -.375, *p* = .045), which indicates more rightward maternal FAA in case of higher infant positive affect scores. Finally, the correlation between maternal FAA scores and PSAS scores during the first still-face episode (*r* = −.287, *p* = 132) and the correlation between maternal affective sensitivity and infant FAA scores during the baseline episode (*r* = −.338, *p* = .068) were no longer significant.

For the third sensitivity analysis, we divided mothers into low- and high-anxiety groups based on their SCL-90 anxiety scores (cut-off score 11) and PSAS scores (median score 73). Results show that mothers and infants in the low-anxiety group based on the SCL-90 had on average more relative left frontal activity across SFP than their counterparts in the high-anxiety group, who had more relative right frontal activity (Fig. S2**.** in Supplementary information). However, the difference was significant only for infant FAA in the second reunion episode (U = 46, *p* = .012). Finally, in the fourth sensitivity analysis no correlations were detected, indicating that our results were not affected by movement and data quality.

## 4 Discussion

The primary purpose of this study was to investigate whether an increase in regulatory demands is associated with altered dyadic FAA and behavior in mother-infant dyads during a double SFP using a smartphone adaptation. Another goal was to study the effect of maternal postnatal anxiety on mother and infant FAA during mother-infant interaction. Consistent with our expectations, we observed a clear still-face effect during our smartphone-adapted still-face paradigm in terms of behavioral data. Infants exhibited a decrease in positive affect and an increase in negative affect during the two still-face episodes (H1a). However, in contrast to our hypotheses, the behavioral still-face effect was not reflected in the neural data (H1b). Our results also did not show a shared pattern of frontal alpha activity between mothers and infants during the double SFP (H1c). Regarding our second aim, we observed that mothers had more rightward FAA during the baseline episode of the double SFP if their infant showed more positive affect (H2a). We also observed a consistent pattern of brain-to-behavior associations among infants, with higher infant negative affect being associated with more infant leftward FAA (H2b) during the still-face episodes. For mothers, higher infant negative affect was associated with more leftward FAA only during the baseline episode. Additionally, we observed that infants of mothers with higher affective sensitivity had more rightward FAA (H2c). Findings for our third aim revealed that maternal postnatal anxiety was associated with maternal FAA (H3a). Specifically, mothers with higher postpartum anxiety showed more rightward FAA during the still-face phase, suggesting negative affectivity and a need to withdraw from the situation. However, these results did not remain significant after sensitivity analysis. We also found that the interaction between maternal postnatal-specific anxiety and infant negative affect did not predict maternal FAA in either still-face episode (H3b). Taken together, our results provide some evidence for intra- and interpersonal brain-to-behavior associations during a double SFP in mothers and infants, suggesting that dyadic behavior is associated with the brain of both mother and infant. However, the direction of effects cannot be determined in the current design.

We demonstrated the feasibility of using EEG hyperscanning to simultaneously measure the neural activity in mothers and infants during a double SFP. In contrast to previous findings, we did not observe significant changes in mother-infant FAA between the free play interaction and still-face episodes (Perone et al., 2020). In our study, we modified the SFP by asking mothers to look at their smartphones during the still-face episodes instead of staring at the child with a neural, “still” face. Moreover, we did not take away toys from the child during the still-face episodes, as is often done in traditional SFP procedures. By including smartphones and access to toys, we created a more naturalistic experimental setup. However, this adaptation could have resulted in a less-stressful experience for the participants and attenuated the negative emotions of infants during still-face episodes (Myruski et al., 2018, Stockdale et al., 2020). Another potential reason for the lack of an effect could be maternal individual differences in approach or avoidance responses to their infant's crying. Our expectations were based on the *valance model* of FAA (Heller, 1993), which suggests that negative affect would be reflected in rightward FAA. We therefore expected mothers to show rightward FAA in response to the still-face episodes (and their crying infant). However, the *approach/avoidance model* of FAA (Kelley et al., 2017) suggests a different possibility: mothers that respond with approach tendencies towards their upset infant (i.e., wanting to comfort their infant) may show leftward FAA, while mothers with avoidance tendencies (i.e., wanting the experiment to just stop) may show rightward FAA. Large inter- and intra-individual differences may exist in approach and avoidance tendencies, based on context, infant responses to the SFP, and maternal personality. For instance, previous research examining maternal postural movements showed that approach/avoidance may not be based on the pleasantness/unpleasantness of infant stimuli, but on the urgency of the stimulus (Hiraoka et al., 2019). Mothers may therefore respond differently, based on their infant's distress signals during the still-face episodes. Future research should therefore ask mothers about their approach/avoidance tendencies during the still-face episodes or combine EEG with measures of respiratory sinus arrhythmia (RSA).

Nevertheless, we observed significant changes in infants’ affective behavior during the still-face episodes, relative to the interaction episodes, which is consistent with previously published data on affective behavior during the SFP (Stockdale et al., 2020, Weinberg and Tronick, 1996; for review see Mesman et al., 2009). This suggests that our modified SFP was effective. Interestingly, we also observed significantly higher infant negative affect in the second reunion episode as compared to the baseline episode. This finding suggests that infants do not return to baseline levels of negative affect after being exposed to maternal still-face episodes. This is in line with literature reporting a partial carryover effect of negative affect from the still-face episode to the reunion episode (Mesman et al., 2009, Weinberg and Tronick, 1996). Given that we used a double SFP which included two still-face episodes reconnecting during the final reunion episode could have been especially stressful and challenging for infants, due to increased regulatory demands (DiCorcia et al., 2016). Our findings on infant’s increasing negative affect, can also potentially be explained by infant’s expectations that a still-face episode will happen again.

Furthermore, our results indicate that mothers and infants seem to respond to each other’s signals, both at a neurophysiological and behavioral level. Interestingly, infants with higher levels of negative affect had more left FAA during the still-face episodes. As described in the literature, left frontal activity is often associated with positive affect, whereas right frontal activity is associated with negative affect (Palmiero and Piccardi, 2017). Within this approach, our findings could be seen as counterintuitive. However, another way to interpret our FAA findings is to look at them from a *motivation perspective*, wherein the right FAA indicates withdrawal-related motivation and the left FAA would indicate approach-related motivation (Harmon-Jones, 2004). Here, some negative emotions, such as anger, may relate to the approach motivational system and may therefore be reflected in leftward FAA (Carver and Harmon-Jones, 2009, Harmon-Jones et al., 2008). Thus, when applying the motivational approach to our findings, we could speculate that the above outcomes in infant brain-to-behavior associations reflect infants' negative affect with approach tendencies, related to increased left frontal activity in infants in still-face episodes. That is, infant frustration and anger in the still-face episodes could reflect an approach tendency associated with infants’ active emotion-regulatory processes geared at re-engaging the mother in social interaction.

We also observed several dyadic brain-to-behavior associations, in which infant behavior was associated with maternal brain responses and in which maternal behavior affected the infant’s brain responses. For mothers, we also observed more leftward FAA in response to higher infant negative affect. Here, the motivation perspective can also be applied: When exposed to her infant’s distress and dysregulated behavior, a mother may feel motivated to soothe her infant, leading to approach tendencies. For infants, we observed more rightward FAA during the baseline episode when their mothers showed higher levels of affective sensitivity. These results may indicate that mothers of infants that show more negative affect or withdrawal tendencies have mothers that are more sensitive toward their affective state. It could be that high affective sensitivity, which may drive mothers to match their infants’ negative state during the still-face paradigm, is not helpful during this situation and creates higher negativity and withdrawal behavior in the infant. However, more research is necessary to confirm this notion.

Next, we examined whether maternal anxiety was associated with mother-infant FAA. Some studies indicate that mothers suffering from anxiety show less involvement and less emotional warmth toward their infants, as well as lower sensitivity in addressing their infant’s needs (Edhborg et al., 2011, Feldman et al., 2009, Reck et al., 2016). Consistent with our expectations, mothers with higher levels of maternal postnatal-specific anxiety symptoms showed more rightward maternal FAA during the first still-face episode, indicating that more anxious mothers may be more likely to experience negative emotional states or a tendency to withdraw during that episode. However, there were no significant associations between infant brain responses and maternal anxiety levels. Our hypothesis about the association between maternal anxiety and infant FAA was based on previous studies showing that infants of depressed mothers exhibit reduced relative left frontal activity (Dawson et al., 1997, Dawson et al., 1999) and infants of mothers with high prenatal anxiety exhibit greater right frontal activity (Field et al., 2003) and prenatal depression (Diego et al., 2006). It could be that maternal postnatal anxiety may be associated with maternal and infant FAA differently than maternal prenatal anxiety.

It is important to note that none of the mothers in our sample qualified for clinical levels of postnatal anxiety. Nevertheless, based on the SCL-90 general anxiety scale, 22 out of 38 mothers had scores indicating some form of elevated anxiety, showing that the mothers in our sample were not completely low in anxiety. For the PSAS scale, we used the “official” cut-off score of 112. However, recent validation studies showed that cut-off scores may differ per country with some countries having (much) lower cut-off scores (Duran and Hakkı, 2020). It would be interesting for future studies to assess whether there are differences between dyads with mothers showing low levels of postpartum-specific anxiety and dyads with mothers with clinical levels of postpartum-specific anxiety. Taken together, our results suggest that maternal anxiety may play a role in maternal, but not infant, neural responses during mother-infant interaction.

There are several strengths and limitations of our study that should be considered. A strength of our study is the naturalistic design of the double SFP. By including smartphones during still-face episodes and letting infants play with toys during SFP, we created a setup that mothers and infants may experience in their daily social interactions. Another strength is the use of EEG hyperscanning method, which allowed us to simultaneously assess brain responses in mothers and infants during a dynamic, interactive context. This approach has the potential to teach us much more about the development of social skills and parenting, than a traditional experimental setup with static stimulation (Wass and Goupil, 2022). A third strength is our evaluation of brain-to-behavior associations, which contributes to the growing knowledge base about associations between dyadic behavior and brain responses.

However, our study has also limitations. One limitation is our inclusion of a broader range of infant ages (6–12 months) than those included in prior SFP studies. Previous research shows that FAA changes over the first two years of life. For example, infants show a resting relative right frontal asymmetry at 9 months and a relative left frontal asymmetry by 14 months that remains stable through 24 months (Fox et al., 2001). Even though, majority of infants included in our study were between 7–9 months, measurements of infant FAA in smaller age windows, where younger or older infants are not included, can provide better insight into the changes in FAA during emotionally challenging situations and build clarity and confidence in interpretation. EEG measurements in children who are closer in age could also limit the possible role of various infant-level factors, such as age-related changes in temperament or motor activity.

Another limitation is that we did not assess the use of smartphones during regular childcare activities by our participating mothers. It has been shown that interruptions to parent-child interactions caused by technology use (called technoference) have been associated with poorer parenting quality (Beamish et al., 2019; Myruski et al., 2018). Infants whose mothers frequently use their phones during caregiving activities may recognize such situations as normative and routine. Thus, the brains of infants who have unavailable parents due to frequent smartphone use may also respond less to the smartphone-adapted still-face paradigm. Prospective research endeavors can explore the differential responses of infants whose mothers frequently engage with smartphones during daily routines in comparison to infants whose mothers utilize smartphones less frequently, with specific focus on their (neural) reactions to the SFP. Furthermore, within our study, we instructed mothers to direct their attention towards a blank screen on their smartphones, thereby establishing a regulated condition across all mother-infant dyads. It would be intriguing to conduct future research that includes a comparative analysis wherein mothers are instructed to either view a blank screen or engage in smartphone usage in a more ecologically valid manner, such as through text messaging.

The relatively low levels of maternal anxiety in our sample is another limitation of our study. The findings we observed here may not generalize to findings in clinical samples. Mother-infant interactions can be seriously impacted by maternal anxiety, depending on its severity and chronicity (Feldman et al., 2009, Prenoveau et al., 2017). Future studies should screen for (postpartum) anxiety and include mothers with varying levels of anxiety symptoms and mothers with an anxiety disorder diagnosis.

Despite the limitations, our results provide a foundation for further research assessing the associations among maternal postnatal anxiety, infant and maternal FAA, and infant socio-emotional behavior during challenging mother-infant interaction tasks. The capacity to regulate emotions adequately in response to stressful situations is an important age-salient task during the first year of life (Sroufe, 1996). Our results complement previous findings that suggest that infants form clear expectations of maternal responsiveness and social interactions at a very early age. As theorized by Tronick et al. (1978), an unresponsive mother violates the infant’s expectations of dyadic face-to-face communication. When infants’ expectations are not met, infants try to re-engage their mothers in social interactions by smiling and looking at their mothers, followed by distress and dysregulation. Another explanation of the still-face effect proposes that during still-face episodes infants lose the scaffolding support of an external regulator (i.e., parent) who can provide optimal levels of stimulation (Field, 1994). Without such relational support, infants’ capacity to sustain attention to the world of people and objects and regulate negative emotions quickly diminishes, resulting in increased negativity and dysregulation that can be measured behaviorally and physiologically. Measurement and assessment of FAA early in life may help identify infants who may be at risk of developing psychopathologies and mother-infant dyads at risk for less optimal interactions. However, even though FAA seems to be related to important dyadic patterns, the direction of the effect is inconsistent and, at times, unexpected. More research is necessary to be able to interpret FAA results in mother-infant interaction and before FAA can be considered as a neural biomarker.

## 5 Conclusion

In summary, we observed a clear behavioral still-face effect in the smartphone-adapted double SFP. However, this was not reflected in the neural data of mothers and infants. Our dyadic brain-to-behavior data suggested, in contrast to our expectations, that infant negative affect was associated with more infant leftward FAA during the still-face episodes. Similarly, mothers had more leftward FAA in response to higher infant negative affect. At baseline, infants had more rightward FAA when their mothers showed higher levels of affective sensitivity. Our results also indicated that maternal postpartum-specific anxiety may play a role in mothers’ neural responses to stressful situations with their infants, suggesting a tendency of mothers with higher anxiety to withdraw from the still-face situation. The results of this study contribute to a better understanding of neural processes during mother-infant social exchange and how these processes are affected by infant affect, maternal anxiety, and the use of a smartphone. Our results also emphasize the need for further research on dyadic neural response during the SFP and the effect of maternal postnatal anxiety on dyadic interactive behavior.

## Declaration of Competing Interest

The authors declare that they have no known competing financial interests or personal relationships that could have appeared to influence the work reported in this paper.
