# Supplementary material for: The association of maternal-infant interactive behavior, dyadic frontal alpha asymmetry, and maternal anxiety in a smartphone-adapted still face paradigm
Source: Dev Cogn Neurosci. 2024 Feb 3;66:101352. doi: 10.1016/j.dcn.2024.101352 (PMC10847859; doi:10.1016/j.dcn.2024.101352)
Supplement: Supplementary file 1 — Supplementary material [file mmc1.docx]

**The association of maternal-infant interactive behavior, dyadic frontal alpha asymmetry, and maternal anxiety in a smartphone-adapted still face paradigm**

**Supplementary Information**

**Table S1.** Intraclass correlation coefficient (ICC) scores of behavioral coding.

| Coded behavior | Intraclass correlation coefficient (ICC) |
| --- | --- |
| Behavioral Sensitivity (Mother) | 0.957 |
| Positive Affect (Mother) | 0.950 |
| Affective Sensitivity (Mother) | 0.953 |
| Warmth (Mother) | 0.898 |
| Object Engagement (Infant) | 0.975 |
| Positive Affect (Infant) | 0.982 |
| Negative Affect (Infant) | 0.966 |
| Flat/Withdrawn (Infant) | 0.896 |

**Table S2.** Mean FAA scores across SFP among girls and boys.

| SFP episode | N | M (SD) | N | M (SD) |
| --- | --- | --- | --- | --- |
|  | Girls FAA |  | Boys FAA |  |
| Baseline | 19 | -.033 (.213) | 17 | -.003 (.216) |
| Still Face1 | 19 | -.023 (.138) | 17 | -.009 (.196) |
| Reunion 1 | 19 | -.041 (.174) | 16 | .025 (.193) |
| Still Face 2 | 17 | -.026 (.202) | 13 | .007 (.256) |
| Reunion 2 | 18 | .009 (.212) | 14 | -.039 (.227) |

**Table S3.** Mean FAA scores across SFP among mothers of girls and boys.

| SFP episode | N | M (SD) | N | M (SD) |
| --- | --- | --- | --- | --- |
|  | Mothers of girls |  | Mothers of boys |  |
| Baseline | 18 | -.014 (.283) | 17 | .132 (.634) |
| Still Face1 | 18 | .023 (.307) | 17 | .082 (.493) |
| Reunion 1 | 18 | -.019 (.343) | 17 | .118 (.683) |
| Still Face 2 | 17 | -.010 (.285) | 13 | .044 (.409) |
| Reunion 2 | 17 | -.044 (.250) | 14 | .087 (.454) |

**Table S4.** Spearman correlations between infant and maternal FAA across SFP.

| Correlation | Baseline | Still Face 1 | Reunion 1 | Still Face 2 | Reunion 2 |
| --- | --- | --- | --- | --- | --- |
| FAA infant x FAA mother | .013 | .271 | -.064 | .041 | -.010 |
| N | 33 | 33 | 33 | 28 | 29 |

**Table S5.** Spearman correlation between mother and infant FAA, SCL-90, PSAS, and IBQ-vsf scores. All participants included.

| Correlation | Baseline | Still Face 1 | Reunion 1 | Still Face 2 | Reunion 2 |
| --- | --- | --- | --- | --- | --- |
| FAA infant x SCL-90 | .119 | .014 | .047 | -.136 | -.069 |
| FAA infant x PSAS | .135 | -.055 | -.023 | .003 | .107 |
| FAA infant x IBQ-vsf NegAffectivity | .061 | -.071 | .274 | -.029 | .172 |
| N | 36 | 36 | 35 | 30 | 32 |
| FAA mother x SCL-90 | -.007 | -.189 | -.077 | -.014 | -.092 |
| FAA mother x PSAS | .027 | **-.380*** | -.021 | -.187 | -.057 |
| FAA mother x IBQ-vsf NegAffectivity | -.031 | -.226 | -.018 | **-.382*** | .030 |
| N | 35 | 35 | 35 | 30 | 31 |

*Correlation is significant at the 0.05 level (2-tailed)

**Table S6.** Multiple regression analyses predicting mother FAA across different SFP episodes with PSAS scores as predictor.

| SFP episode | R^2^ | β | t |
| --- | --- | --- | --- |
| Baseline | .000 | -.011 | -.064 |
| Still Face 1 | .044 | -.209 | -1.23 |
| Reunion 1 | .000 | -.001 | -.003 |
| Still Face 2 | .028 | -.166 | -.892 |
| Reunion 2 | .002 | -.044 | -.235 |

**Table S7.** Multiple regression analyses assessing effects of maternal PSAS and infant negative affect interaction on mother FAA during still-face episodes (SF1 and SF2).

| Predictors | R^2^ | β | t |
| --- | --- | --- | --- |
| Model SF1 | .066 |  |  |
| PSAS |  | -.497 | -1.164 |
| Infant Negative Affect |  | -.716 | -.679 |
| Interaction PSASxInfant Negative Affect |  | .822 | .756 |
| Model SF2 | .031 |  |  |
| PSAS |  | -.090 | -.189 |
| Infant Negative Affect |  | .159 | .121 |
| Interaction PSASxInfant Negative Affect |  | -.230 | -.160 |

**Table S8.** Spearman correlation between mother and infant FAA, SCL-90, PSAS, and IBQ-vsf scores. Participants with complete data from all SFP episodes (N=29).

| Correlation | Baseline | Still Face 1 | Reunion 1 | Still Face 2 | Reunion 2 |
| --- | --- | --- | --- | --- | --- |
| FAA infant x SCL-90 | -0.23 | -.055 | .034 | -.133 | -.146 |
| FAA infant x PSAS | .058 | -.143 | .022 | -.028 | .092 |
| FAA infant x IBQ-vsf NegAffect | .033 | -.121 | .277 | -.037 | .182 |
| FAA mother x SCL-90 | -.004 | -.130 | -.074 | .038 | -.053 |
| FAA mother x PSAS | -.042 | -.287 | -.028 | -.139 | .011 |
| FAA mother x IBQ-vsf NegAffect | -.015 | -.336 | -.018 | **-.383*** | .027 |

*Correlation is significant at the 0.05 level (2-tailed)

**Table S9.** Spearman correlation between infant and maternal FAA and infant positive and negative affect. Participants with complete data from all SFP episodes (N=29).

| Correlation | Baseline | Still Face 1 | Reunion 1 | Still Face 2 | Reunion 2 |
| --- | --- | --- | --- | --- | --- |
| Infant FAA x Infant positive affect | -.270 | -.191 | -.086 | -.331 | -.172 |
| Infant FAA x Infant negative affect | .258 | **.476**** | **.396*** | **.538**** | -.076 |
| Mother FAA x Infant positive affect | **-.375*** | -.241 | -.013 | .157 | -.137 |
| Mother FAA x Infant negative affect | .210 | .041 | .077 | -.010 | -.224 |

**Correlation is significant at the 0.01 level (2-tailed)

*Correlation is significant at the 0.05 level (2-tailed)


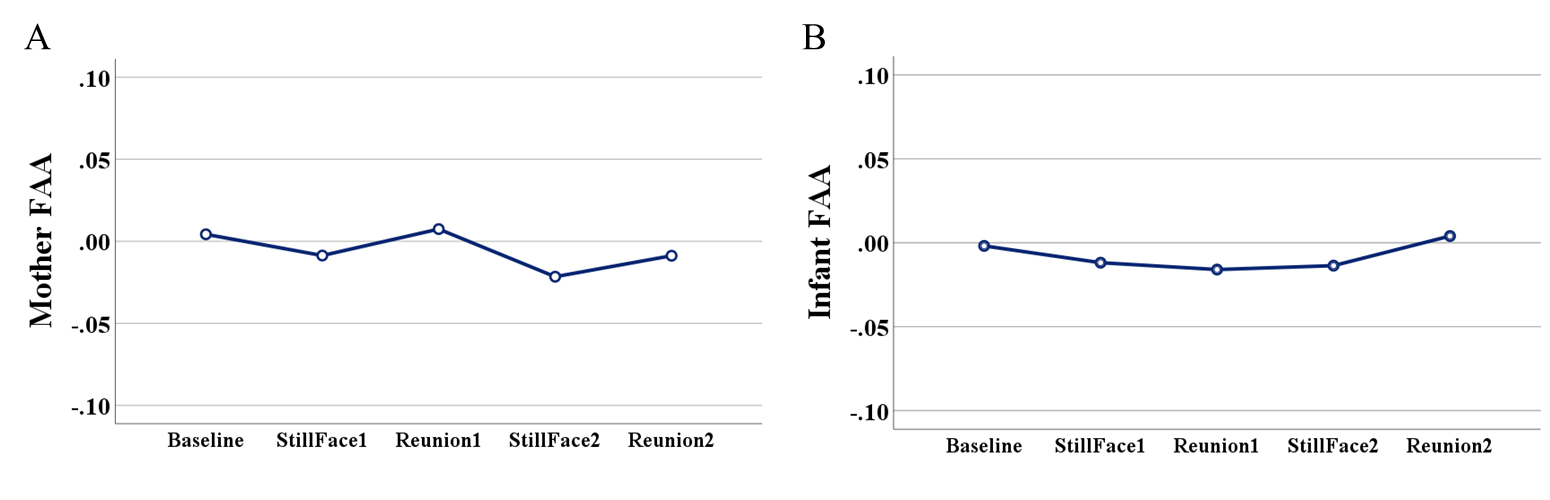


**Figure S1**. FAA across SFP episodes measured with EEG. FAA scores across each SFP task for (A) infants and (B) mothers (N=29).


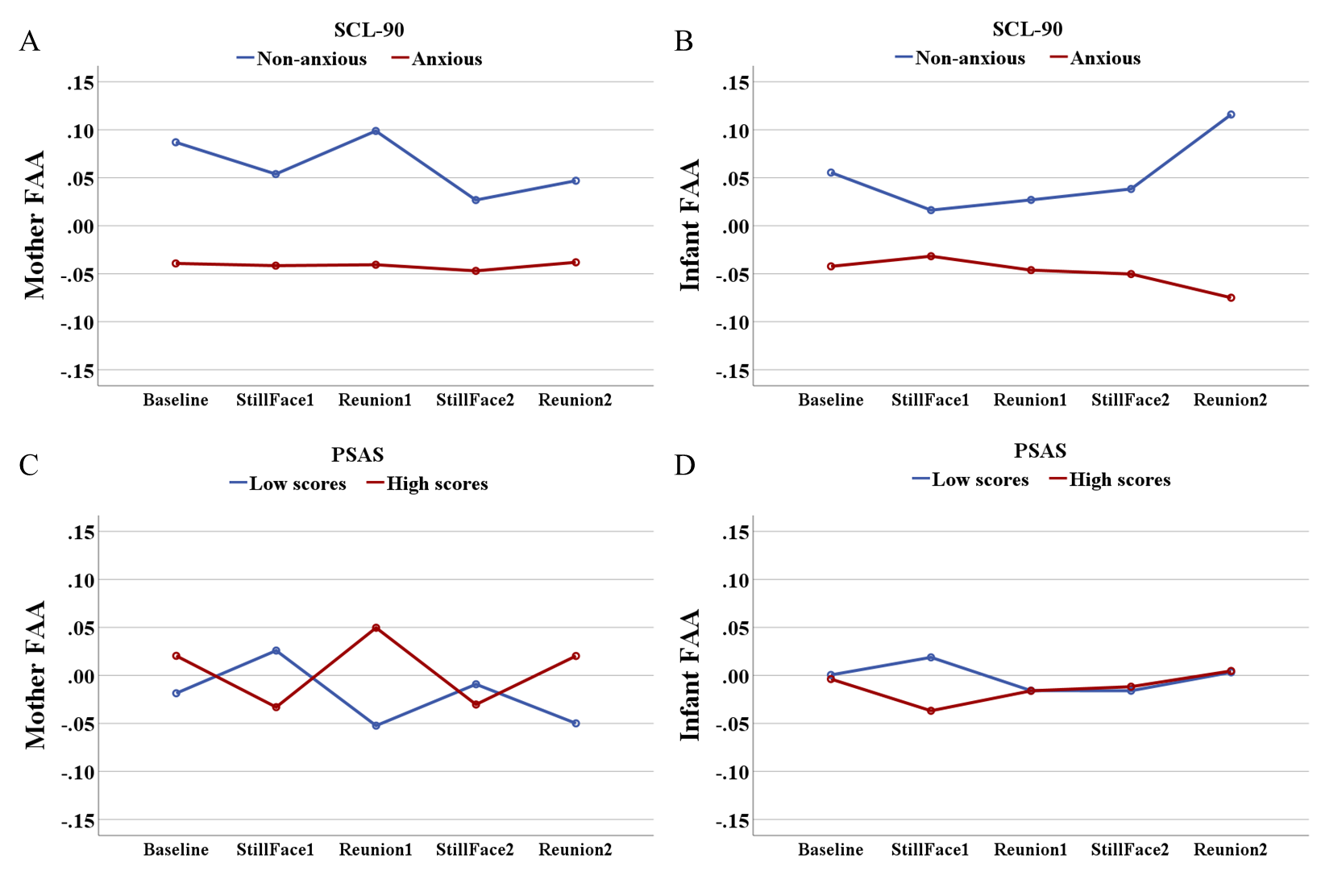


**Figure S2.** Differences in average FAA scores across SFP episodes based on maternal anxiety scores. Average (A) maternal (Non-anxious N=10, Anxious N=19) and (B) infant (Non-anxious N=12, Anxious N=17) FAA scores based on maternal SCL-90 cut-off scores. Average (C) maternal (Low scores N=12, High scores N=17) and (D) infant (Low scores N=13, High scores N=16) FAA scores based on maternal PSAS median scores.
